# Population based Air Pollution Exposure and its influence factors by Integrating Air Dispersion Modeling with GIS Spatial Analysis

**DOI:** 10.1038/s41598-019-57385-9

**Published:** 2020-01-16

**Authors:** Xiaoya Dong, Xiuge Zhao, Fen Peng, Danlu Wang

**Affiliations:** 1 School of Education Science, Hunan First Normal University, Changsha, 410205 China; 2 State Key Laboratory of Environmental Criteria and Risk Assessment, Chinese Research Academy of Environmental Sciences, Beijing, 100012 China; 3 School of Architecture, Changsha University of Science and Technology, Changsha, 410076 China

**Keywords:** Environmental sciences, Environmental sciences, Environmental sciences, Risk factors, Risk factors

## Abstract

Air pollution is a major environmental health problem. The study of interaction between air pollution and human will benefit to the human health and well-being of community. Both a model for assessing population relative risk of air pollution exposure (MAPRRAPE) and air pollution concentration methods were applied in a case study to determine the optimal method in evaluating risk of population exposure to Sulfur Dioxide (SO_2_). The framework for building the MAPRRAPE was described in detail. Then, the spatial patterns of population by demographic characteristics exposed to SO_2_ from industrial, vehicle, and the mixture of industrial and vehicle pollution sources, as well as an in-depth quantitative investigation using correlation analysis were studied for further source appointment. The results showed that the MAPRRAPE was more reliable than air pollution concentration model in determining population exposure risks by demographic characteristics. The high risk areas of whites exposed to SO_2_ were larger than blacks and the other races due to a large number of whites, and other age groups exposed to SO_2_ were larger than children and the old people. In addition, the correlation analyses showed that the relative risks of population by demographic characteristics exposed to SO_2_ had a more significant correlation with vehicle pollution source than industrial pollution source. The results of source appointment thus demonstrated that vehicle pollution source was the main pollution source. This study suggests that there is a clear need for the implementation of programs and services that will reduce population exposed to air pollution with focusing on densely populated areas for an ultimate improvement of community health status and the environmental conditions.

## Introduction

Air pollution is a major environmental health problem in both global developed and developing countries. Thus carrying out study about air pollution exposure assessment and its influence factors will benefit the public health and well-being of community, mostly susceptible population groups as the result of such intervention disseminated would inevitably enhance personal response in reducing exposure to pollutants, or particular harmful agent^1–4^. In addition, the adverse effects of exposure to air pollutants prevail in both large and medium-sized cities^5,6^. Therefore, accurate estimates of population exposure to air pollutants at a high level in detail are necessary for assisting the intervention of decision-maker and residents to management and control the exposure from air pollution.

In an effort to achieve the reduction or elimination of human exposure to air pollution, researchers have been focusing on two key exposure assessment methods: (1) one method that quantifies exposure by determining the concentrations of air pollutants releasing to the environment, such as proximity models^7,8^, air dispersion models^9,10^, land use regression models^11,12^, and interpolation models^13,14^; (2) the another method is an air pollution exposure model taking population distribution characteristics into consideration^15,16^. Kousa, *et al*.^15^ established population space mobile characteristics-based mean air pollution exposure model to estimate individual air pollution exposure. Beckx, *et al*.^17^ proposed a dynamic activity-based population modeling approach to reveal the substantial differences between the static and dynamic exposure for evaluating population exposure to PM_10_ and PM_2.5_. Although these models are characterized with more accuracy and theoretical reliability for estimating air pollution exposure, the collection of individual exposure data in a large region is time-consuming and costly. Wong, *et al*.^18^ developed an analysis on population exposure to urban heat island intensity by socio-demographic characteristics at a spatial scale of 120 m. Thus, it is possible to develop accurate estimates of exposure to air pollutants at a high spatial resolution.

In addition, estimations of sources contributions to ambient air pollution need to be established to better comprehend the behavior and advection of ambient air pollution for guiding susceptible population groups far away from pollution sources and determining exposure–response relationships from health effects. Air pollutants are primarily emitted from a few point and line sources such as various industrial processes (e.g., smelters, coal–fired power plants) and the emission of exhaust from motor vehicles with low–grade diesel fuel^19^. A particular interest of this study is that it explores the issues of sulfur dioxide (SO_2_). Recent studies found that low concentrations of SO_2_ are still possibly associated with adverse health effects^20^, even peak is further related to mortality^21^. The rates of preterm births also increased among women living in the vicinity of a coal-burning power plant with higher SO_2_ emissions in Croatia^22^.

In the light of the above, the study will integrate air dispersion model, Geographical Information System (GIS), Population Dynamic Mapping Model (PDMM) with spatial interpolation technique to establish a Model for Assessing Population Relative Risk of Air Pollution Exposure (MAPRRAPE) at a high spatial resolution. Then, the study will apply the MAPRRAPE and concentration model in a case study area to determine the advantage of developed framework in estimating human exposure risk. In addition, it will analyze the risk of exposure to SO_2_ with respect to socio-demographic characteristics and influence factors in the specific land use context of case study area.

## Methods

### Study area and data sources

**Figure 1:**
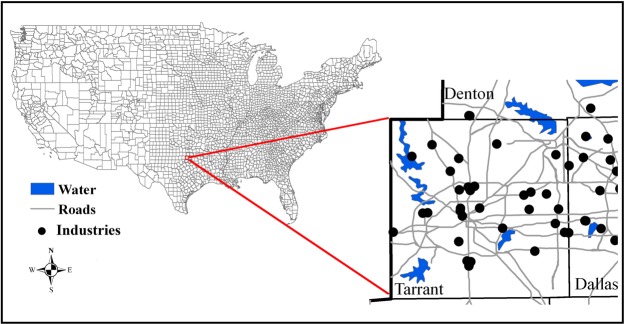
Study area.

A case study area is selected to develop a risk analysis of air pollution exposure, which covers an area of 2324 km^2^ with 1033 census blocks, with a number of highways, interstate, and state roads. The population density is 647 persons per square kilometer. Tarrant County is located at latitude 32.57°N–33.00°N, longitude 97.04°W–97.53°W. Tarrant County is a county equivalent area found in Texas, USA (Fig. 1). The county government of Tarrant is found in the county seat of Fort Worth. In addition, the factory locations, road network distribution maps, demographic data, and land use data in the case study area were collected^23–25^. The SO_2_ emission data in Tarrant County was extracted from the 2000 National Emission Inventory (NEI)^26^ and it was estimated that a total of 237.79 tons of SO_2_ was emitted from 33 industrial point sources and an additional 929 tons from vehicles on major roads (e.g. highways, major local streets) in Tarrant County in the year 2000. Meteorological data including near-surface measurement and upper-air sounding data were extracted from the Integrated Surface Hourly database and the Radiosonde database at the National Climatic Data Center^27^ and the National Oceanic and Atmospheric Administration^28^, respectively. The near-surface measurements (e.g. temperature, precipitation) were conducted with the anemometer height of 2 m at hourly intervals from one meteorological observation site (elevation: 134.1 m). The upper-air sounding data were measured at 12-hr interval at one meteorological site (elevation: 196.3 m). Two 1° U.S. Geological Survey (USGS) digital elevation datasets at a scale of 1:250,000 were utilized to represent the topographic characteristic of study area. The elevation in the study area ranged from 100 m to 262 m with a mean elevation of 155.7 m. The block-level population data was extracted from the last full U.S. Census in 2000^29^.

### The framework of MAPRRAPE

**Figure 2:**
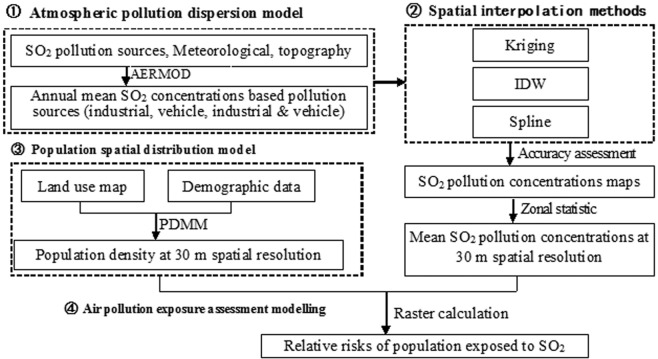
The flowchart of MAPRRAPE.

The MAPRRAPE is mainly summarized as four steps and described in Fig. 2. Step one is atmospheric pollution dispersion model. The discrete annual mean SO_2_ concentrations from industrial, vehicle, combined industrial and vehicle pollution sources were obtained by the atmospheric pollution dispersion model of American Meteorological Society/Environmental Protection Agency Regulatory Model (AERMOD) in the previous study^30^. Step two is to select the optimal spatial interpolation method. It compared Kriging, Inverse Distance Weighted (IDW) and Spline interpolation methods and found that IDW was the most appropriate spatial interpolation method to obtain the spatial patterns of SO_2_ concentrations^31^. Step three indicates that the spatial distributions of population by different demographic groups are obtained using PDMM method. Step four uses the MAPRRAPE to model relative risks of population exposed to SO_2_ pollution.

**Figure 3:**
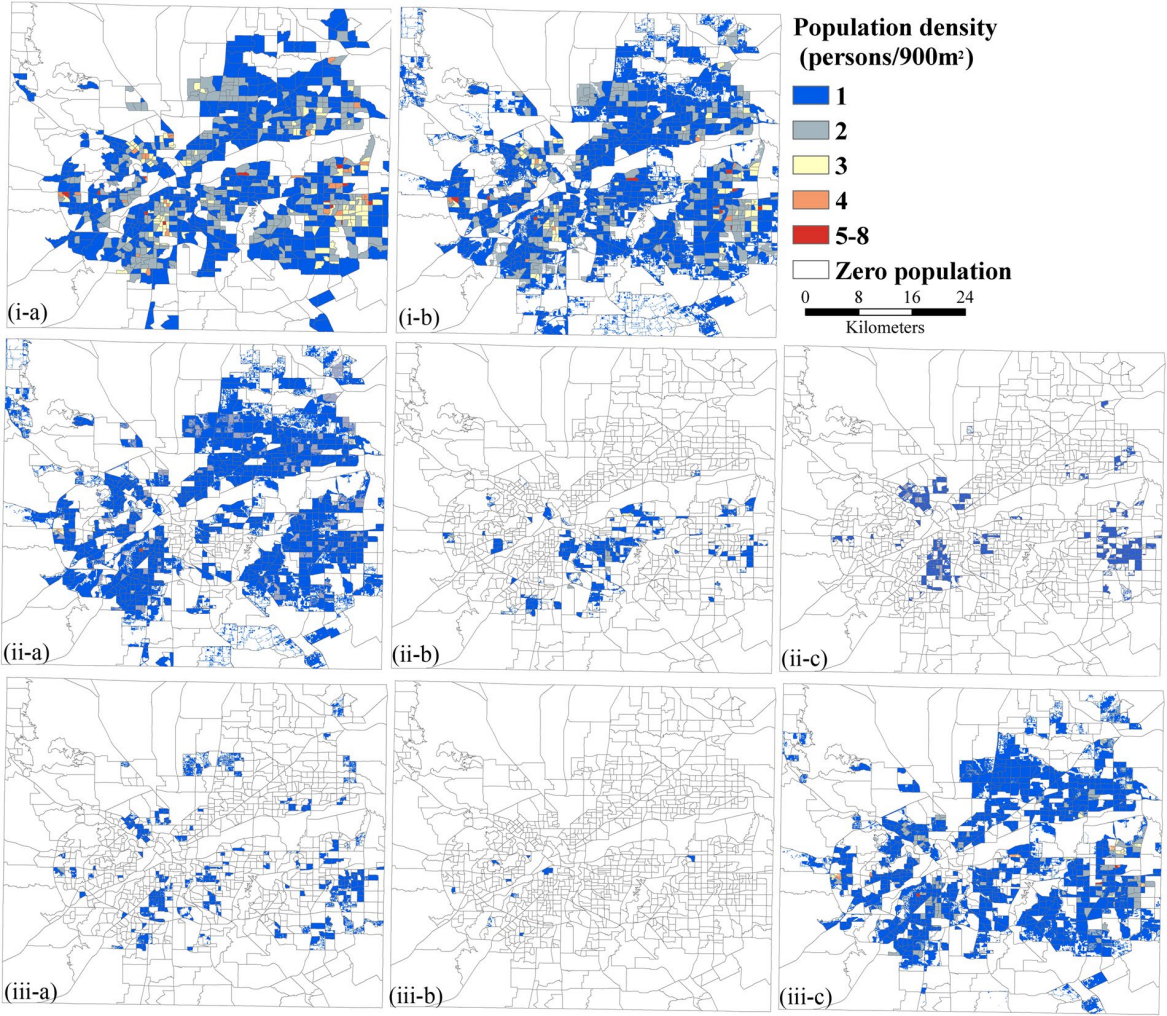
The spatial patterns of population densities. (i-a) block scale, (i-b) 30 m spatial scale, (ii-a) white, (ii-b) black, (ii-c) the other races, (iii-a) child, (iii-b) the elderly, (iii-c) the other age groups.

Specifically, the PDMM method was used to simulate spatial distribution of population density in different homogenous zones based on both areal weighting interpolation and an urban-classification method^32^. Empirical sampling provides a proportional density fraction used as a weighted value representing each urban class and is based upon land use and land cover (LULC) data at 30 m spatial resolution. The ArcMap extension module ‘dasymetric mapping’ is used to obtain population density, which can automate the areal interpolation process within a GIS framework^33^. Figure 3 shows spatial patterns of population density at 30 m spatial resolution by age (child, the elderly, the other age groups) and race characteristics (white, black, the other races including Native American, Pacific Islander, Asian, Hispanic).

The relative risks of population exposed to SO_2_ pollution from industrial, vehicle, combined industrial and vehicle pollution sources is estimated using the MAPRRAPE, which is shown in Eq. (1)^34^.

1 $${R}_{ij}=(po{p}_{i}\times {C}_{ij})/((\mathop{\sum }\limits_{i=1}^{n}po{p}_{i}\times {C}_{ij})/n)$$

where *i* indicates grid, *j* denotes air pollution sources, *R*_*ij*_ represents grid *i*’s relative risk of population exposed to the pollution source *j*, *pop*_*i*_ is the population of grid *i*, *C*_*ij*_ denotes the air pollution concentration of grid *i* from the pollution source *j*, *n* denotes the total grid numbers in a spatial statistical/administrative unit with demographic data.

## Results

### The comparison of air pollution exposure assessment methods

**Figure 4:**
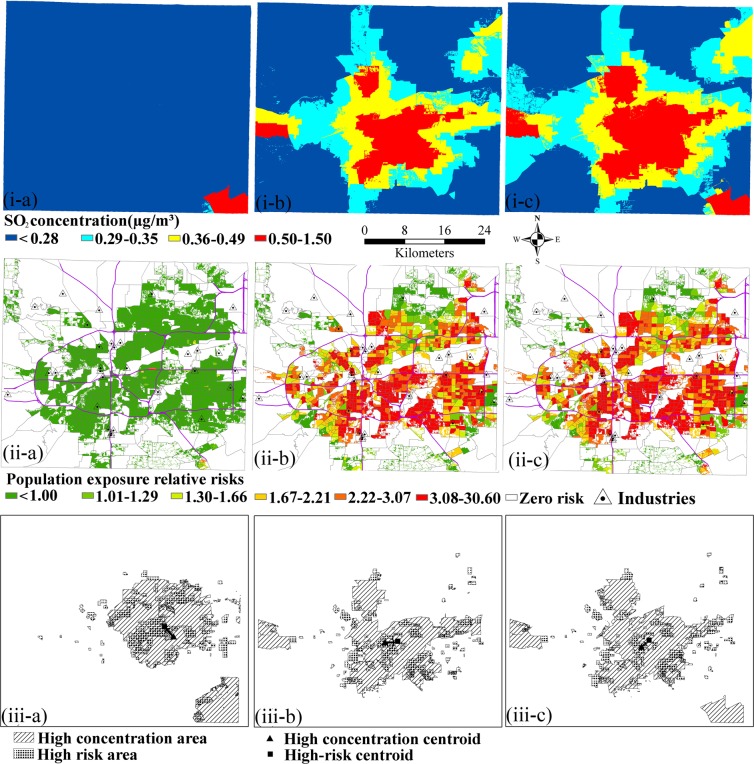
The spatial patterns and centroid shifts of high risk areas (**a**–**c**) denotes industrial, vehicle, combined industrial and vehicle pollution sources.

Figure 4(i-a, i-b, i-c) shows the spatial distributions of SO_2_ annual mean concentrations from the industrial, vehicle, combined industrial and vehicle pollution sources. The concentration was broken into four ‘natural break’ levels using the unit ‘μg/m^3^’ with categories of 0.00–0.28 (Low), 0.29–0.35 (Low-Med), 0.36–0.49 (Med), and 0.50–1.50 (High) based on the natural break values of the concentrations. The areas of high SO_2_ concentration (>0.49 µg/m^3^) were mainly located in the southeastern parts of case study area, while the other areas had a concentration of less than 0.28 µg/m^3^. With respect to vehicle pollution source, it is obvious that areas of high SO_2_ concentration were unevenly distributed in the vicinity of highways and the intersection of main roads. When industrial and vehicle pollution sources were combined, the areas of high SO_2_ concentration were located significantly in the vicinity of main roads around the central area. In addition, it was found by the comparison to Fig. 4(i-a), (i-b), and (i-c) that the spatial pattern of SO_2_ concentration from the mixture of industrial and vehicle pollution sources was an integration of the spatial patterns from industrial and vehicle pollution sources, even had an expansion of high SO_2_ concentration areas. Figure 4(ii-a, ii-b, ii-c) show the relative risks of population exposed to SO_2_ from the industrial, vehicle, combined industrial and vehicle pollution sources. The relative risk less than 1.0 indicates that the risk of people exposed to SO_2_ concentration in this grid is less than the mean risk of exposure to SO_2_ concentration in the county. The more relative risk value is, the higher people exposed to SO_2_ concentration is. The relative risk was broken into six ‘natural break’ levels with categories of 0.00–1.00 (Low), 1.01–1.29 (Low-Med), 1.30–1.66 (Med), 1.67–2.21 (High-Med), 2.22–3.07 (High), and 3.08–30.60 (Extreme High) based on the natural break values of the concentrations. Figure 4(ii-a) shows that the high relative risk areas of total population exposure to SO_2_ were mainly located in the central, eastern and southeastern parts of case study area. The high-risk areas in the southeast were mainly caused by high SO_2_ concentrations, whereas the other areas were more likely to be affected by high population density. The high relative risk areas of total population exposure to SO_2_ were mainly located in the central and eastern parts of case study area, and shown in Fig. 4(ii-b). The decreased risk from vehicle pollution source in the southeastern area compared to industrial pollution source was caused by low SO_2_ concentrations. Figure 4(ii-c) shows that the high relative risk areas of total population exposure to SO_2_ were mainly located in the central and eastern parts of case study area. Then, the top 10% of SO_2_ concentrations and relative risks at 30 m spatial resolution was extracted to develop a centroid shift analysis, which is shown in Fig. 4(iii-a, iii-b, iii-c). It shows that there is a significant shift of centroids between high concentration and high relative risk areas. In addition, the distance of centroid shift between high concentration and high relative risk areas were measured for further analyzing the difference of high risk areas from the two risk assessment methods. The centroid distances of the two high risk areas are 2.94, 2.67, 2.22 km based on the industrial, vehicle, combined industrial and vehicle pollution sources.

### The risk analysis from industrial pollution source by race and age

**Figure 5:**
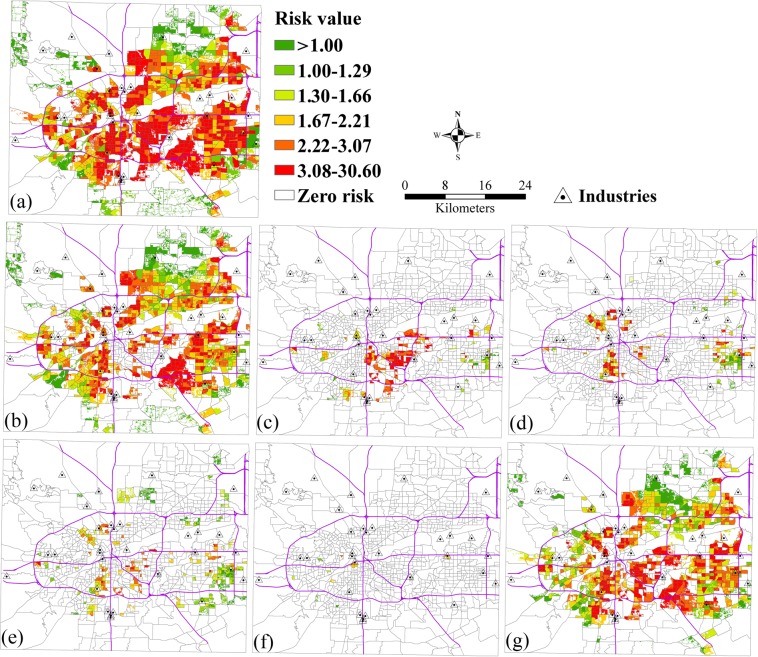
The relative risks of exposure to SO_2_ from industrial pollution source by race and age. (**a**) Total population, (**b**) White, (**c**) Black, (**d**) Other races (Native American, Pacific Islander, Asian, Hispanic), (**e**) Children, (**f**) Elderly, (**g**) Other groups.

The relative risks of population by race and age exposed to SO_2_ pollution from industrial pollution sources is shown in Fig. 5. Specifically, Fig. 5(b–d) shows the spatial patterns of relative risks of whites, blacks, and the other races exposed to SO_2_. It shows that the high risk areas of whites exposed to SO_2_ were larger than blacks and the other races due to a large number of whites. The high relative risks for blacks mainly focused on the central case study area, and the risks for the other races were all less than 3.08. Figure 5(e–g) shows the spatial patterns of relative risks of children, the elderly, and the other age groups exposed to SO_2_. It shows that the high risk areas of other age groups exposed to SO_2_ were larger than children and the old people due to a large number of population. Few relative risks of the elderly exposed to SO_2_ were found in the case study area.

### The risk analysis from vehicle pollution source by race and age

**Figure 6:**
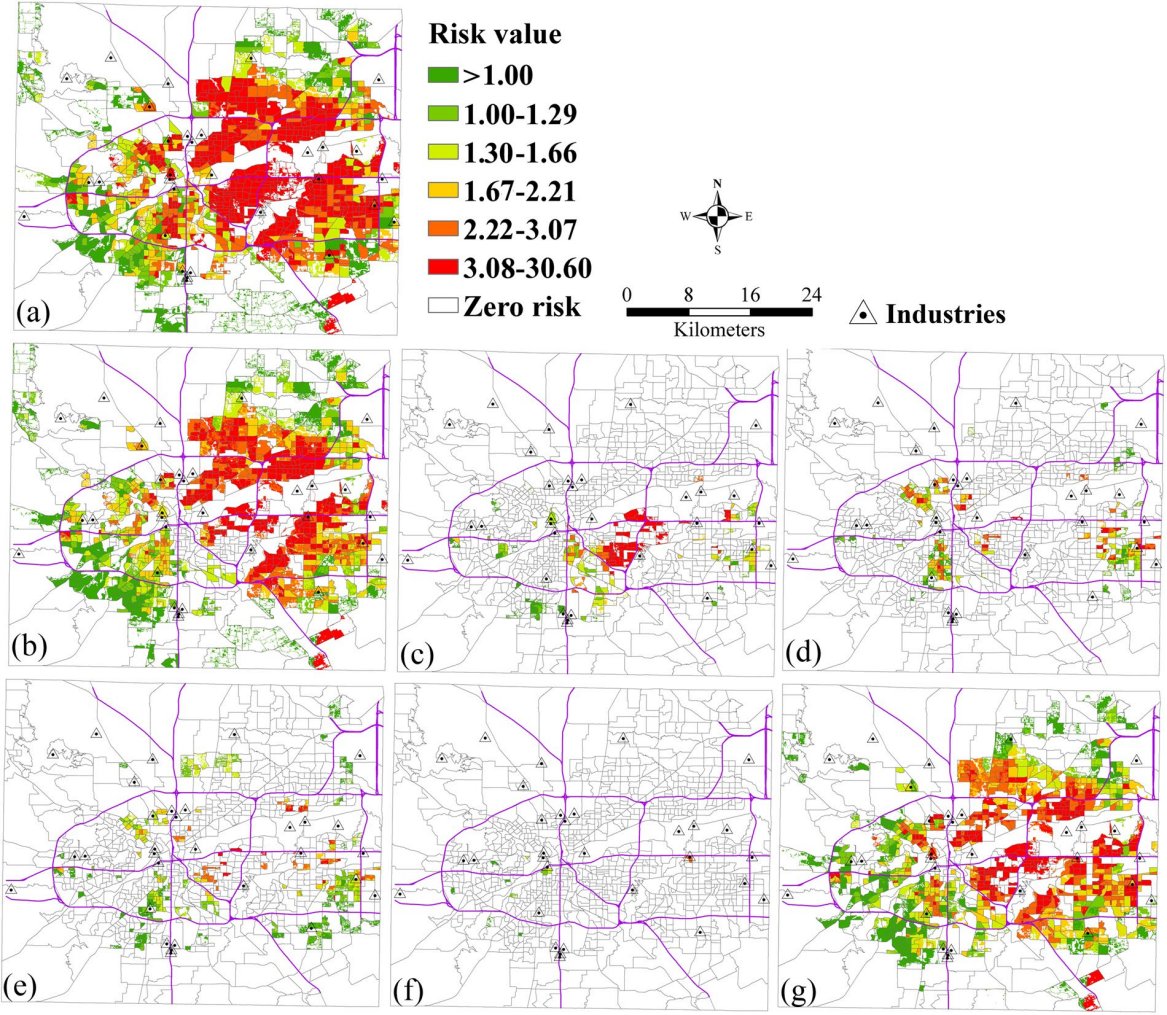
The relative risks of exposure to SO_2_ from vehicle pollution source by race and age. (**a**) Total population, (**b**) White, (**c**) Black, (**d**) Other races (Native American, Pacific Islander, Asian, Hispanic), (**e**) Children, (**f**) Elderly, (**g**) Other groups.

The relative risks of population by race and age exposed to SO_2_ pollution from vehicle pollution source is shown in Fig. 6. Figure 6(b–d) show the spatial patterns of relative risks of whites, blacks, and the other races exposed to SO_2_. These show that the high-risk areas of whites exposed to SO_2_ were larger than blacks and the other races. The high relative risks for blacks mainly focused on the central case study area, and the risks for the other races were almost all less than 3.08. Figure 6(e–g) show the spatial patterns of relative risks of children, the elderly, and the other age groups exposed to SO_2_. These show that the high-risk areas of other age groups exposed to SO_2_ were larger than children and the old people due to a large population base. Few relative risks of the elderly exposed to SO_2_ from vehicle pollution source were similarly found in the case study area.

### The risk analysis from the mixture of industrial and vehicle pollution sources by race and age

**Figure 7:**
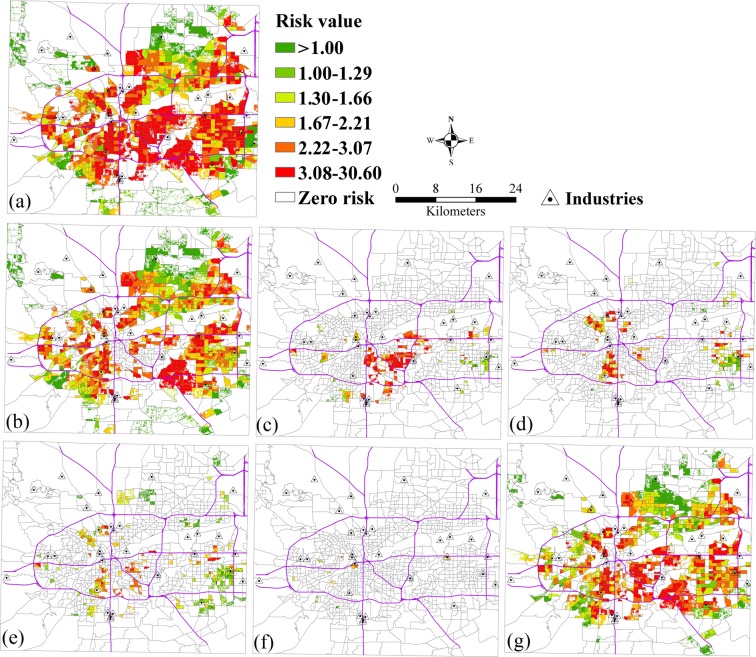
The relative risks of exposure to SO_2_ from the mixture of industrial and vehicle pollution sources by race and age. (**a**) Total population, (**b**) White, (**c**) Black, (**d**) Other races (Native American, Pacific Islander, Asian, Hispanic), (**e**) Children, (**f**) Elderly, (**g**) Other groups.

The relative risks of population by race and age exposed to SO_2_ pollution from the mixture of industrial and vehicle pollution sources is shown in Fig. 7. The spatial patterns of relative risks from the mixture of industrial and vehicle pollution sources had a similarity with vehicle pollution source. Figure 7(b–d) show the spatial patterns of relative risks of whites, blacks, and the other races exposed to SO_2_. These show that the high risk areas of whites exposed to SO_2_ were larger than blacks and the other races. The high relative risks for blacks mainly focused on the central case study area, and the risk areas for the other races were few. Figure 7(e–g) show the spatial patterns of relative risks of children, the elderly, and the other age groups exposed to SO_2_. These show that the high risk areas of other age groups exposed to SO_2_ were larger than children and the old people.

### The influence factors of exposure risk by demographic characteristics

**Figure 8:**
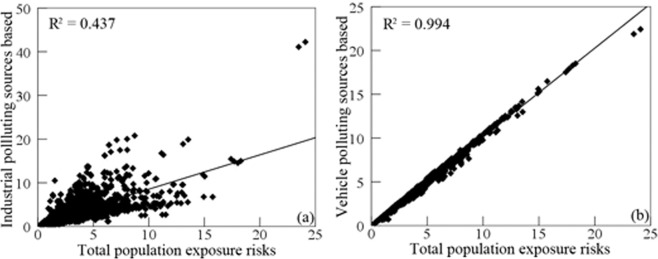
Scatter plots of the relative risks of population exposed to SO_2_ from industrial and vehicle pollution sources. (**a**) industrial pollution source, (**b**) vehicle pollution source.

**Figure 9:**
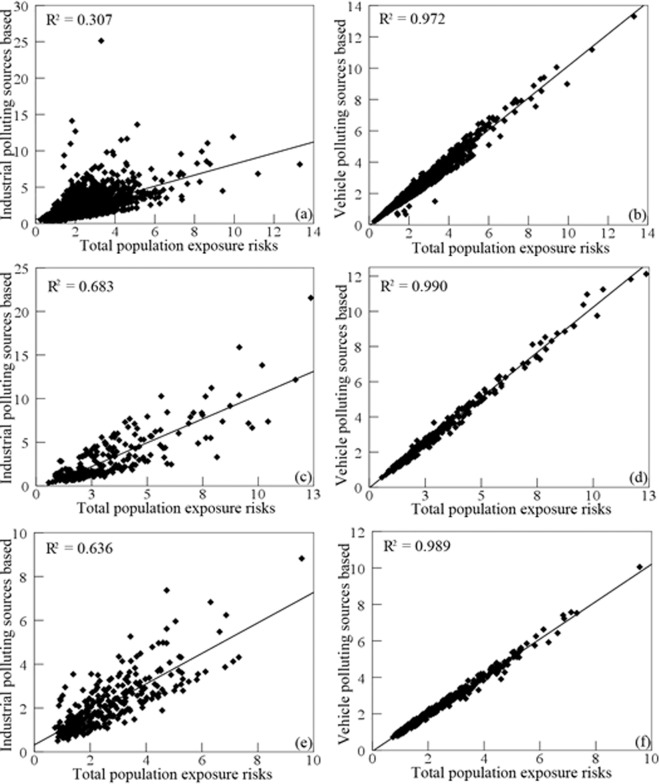
Scatter plots of the relative risks of population by race exposed to SO_2_ from industrial and vehicle pollution sources. Industrial pollution source: (**a**) white, (**c**) black, (**e**) the other races; vehicle pollution source: (**b**) white, (**d**) black, (**f**) the other races.

**Figure 10:**
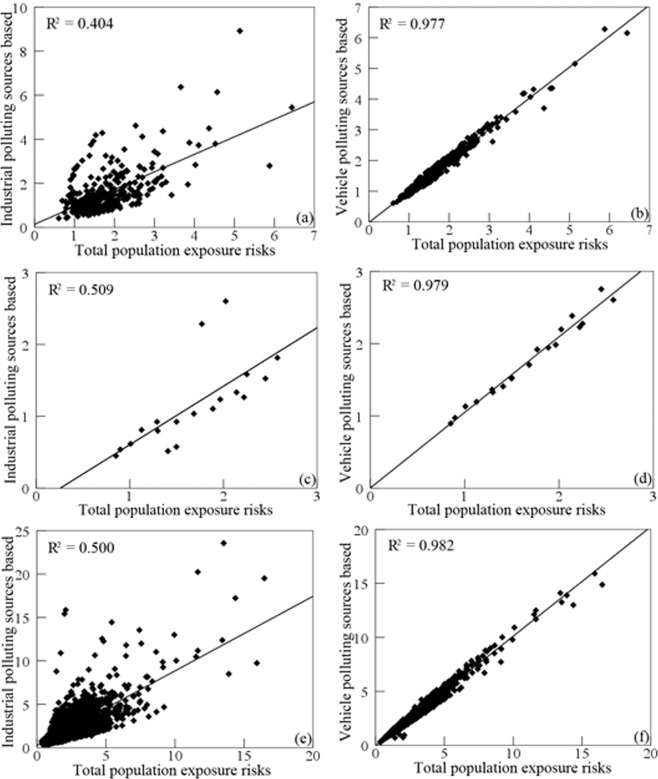
Scatter plots of the relative risks of population by age exposed to SO_2_ from industrial and vehicle pollution sources. Industrial pollution source: (**a**) child, (**c**) the elderly, (**e**) the other age groups; vehicle pollution source: (**b**) child, (**d**) the elderly, (**f**) the other age groups.

The influence factors for relative risks of population exposed to SO_2_ from the industrial and vehicle pollution sources was explored using scatter plot and Pearson correlation coefficient. Figure 8 shows that there was a significant positive linear correlation between the vehicle pollution source and total population exposure risks, and Pearson correlation coefficient was 0.994 with p-value < 0.05. Figure 9 shows that the relative risks of population by race exposed to SO_2_ had a more significant correlation with vehicle pollution source than industrial pollution source, due to Pearson correlation coefficient based on vehicle pollution source were 0.972, 0.990, and 0.989 for whites, blacks, and the other races. Similarly, Fig. 10 shows that the relative risks of population by age exposed to SO_2_ had a more significant correlation with vehicle pollution source than industrial pollution source, and Pearson correlation coefficient based on vehicle pollution source were 0.977, 0.979, and 0.982 for child, the elderly, and the other age groups. Therefore, it is obvious that vehicle pollution source contribution to population by demographic characteristics exposed to SO_2_ was more significant than industrial pollution source.

## Discussion

The study firstly demonstrated that the MAPRRAPE in evaluating risk of population exposed to air pollution was more reasonable than air pollution concentration, as the MAPRRAPE was performed basing upon the interaction between air pollution and human. In addition, it needs to be pointed out that the concentration of an air pollutant is the amount of the pollutant per unit volume of air, which presents a physical characteristic of the environment^35^. Exposure refers to that the body surface keeps contact with air pollutant, specifically it requires the presences of a person and an air pollutant concentration simultaneously^36,37^. Therefore, air pollution concentration only determines the pollution situation but makes no assumption about whether a person is exposed to the pollution or not. Exposure estimate is a more accurate measure on human contact with pollution by comparison with air pollution concentration, and thus it is a critical component of health risk assessment^16^. In addition, the MAPRRAPE provides an effective framework to estimate the extent to which people are exposed to SO_2_ pollution.

Considering the important role and severe pollution status of SO_2_, many efforts should be made to locate the area of the high SO_2_ pollution especially in the highly populated regions^38^. This study hereby develops the MAPRRAPE to evaluate risk of population exposed to SO_2_. Khaniabadi *et al*.^39^ used the relative risk which is the possibility of developing a sickness as a result of the exposure to a pollutant^40^ to assess the health impacts of exposure to SO_2_. However, this approach used in this study focuses on total SO_2_ pollution without considering the exposure to SO_2_ from different pollution sources, which is what actually occurs. On basis of this, our study evaluated the interactions between SO_2_ pollution from different pollution sources. The application of technical methods can help ascertain which sulfur emissions of pollution sources need to be urgently decreased in a highly populated area. The health risk assessment for the SO_2_ exposure across the whole China has not been performed yet, which was mainly attributed to the limitations in the availability of high-resolution SO_2_ data^41^. Our study developed a model to study the relative risk at 30 m spatial resolution, and can be applied to other areas with severe SO_2_ pollution. The study helps policymakers to locate the high-risk areas of susceptible population exposed to air pollution with a high spatial resolution.

An in-depth quantitative investigation was used to analyze the industrial and vehicle pollution sources contribution to population by demographic characteristics exposed to SO_2_. It found that the relative exposure risks had a more significant correlation with vehicle pollution source than industrial pollution source. Bozkurt *et al*.^42^ found that traffic is one of the most important sources of air pollution in Düzce. Ielpo *et al*.^43^ found that SO_2_ was mainly associated to industrial sources present in the South Italy. Our study area has an intensive vehicular traffic with the 20, 30, 820 Interstate roads and 81, 287 State roads passing through the city. According to SO_2_ emission data, a total of 237.79 tons of SO_2_ was emitted from 33 industrial point sources and an additional 929 tons from vehicles on major roads. The total emission rate of industrial point sources is 7.54 g/s, and the total emission rate of traffic line sources is 29.45 g/s. For this reason, traffic is one of the most important sources of SO_2_ in Tarrant County. When air pollution concentration from the vehicle pollution source was higher than the industrial pollution source, the simulated relative exposure risks were more influenced by air pollution concentration. Conversely, low SO_2_ concentration from the industrial pollution source gave rise to population exposure risk be more dependent on the demographic data. Transportation still plays an important role in economic growth, social and economic cohesion and development, and delivery/transport of industrial devices or products/goods mostly in remote areas that lead to high consumptions of fuels. The MAPRRAPE is therefore demonstrated again that it is very important and indispensable framework for risk assessment of air pollution from a human health perspective.

In the study, AERMOD was used to simulate air pollution concentrations from industrial, vehicle, and the mixture of industrial and vehicle pollution sources for source appointment, whereas it is difficult to differentiate the pollution sources using air pollution monitoring data. In addition, the finding of the study suitably deduces that the increased air pollution concentrations resulted from both industrial and vehicle pollution sources are ascribed to the additive effect of both sources, especially vehicle pollution source. It needs therefore more support and attention be given to implementation of tighter vehicle emission standards in the future decision-making. The developed analysis framework can also serve as a supporting tool for focusing on the high-level evaluation of traffic-related and industrial-related air pollution using limited and aggregate spatial and traffic data.

## Conclusion

In summary, both MAPRRAPE and air pollution concentration methods were able to evaluate risk of population exposure to SO_2_, but the MAPRRAPE was more reliable than concentration model in determining population exposure risks by demographic characteristics. There were differences between the risk of population exposure for different race and age. The high risk areas of whites exposed to SO_2_ were larger than blacks and the other races, and other age groups exposed to SO_2_ were larger than children and the old people. Population, as well as industrial and vehicle pollution, were main contributor to SO_2_ exposure in study area, among which vehicle pollution was the most important regardless of race and age. The study provides decision-makers with the identification of vehicle pollution source resulting in a wide scope of high exposure risk, sheds light on understanding the spatial distribution with respect to population exposed to each source by demographic characteristics. Implementing sustainable reduction strategy of air pollution in the case study area will ward off the health hazards associated with breathing in air pollution. Strategies that reduce the risk of exposure to air pollution should be implemented such as vehicle exhaust emissions limits and ambient air quality legislation and/or enforcement.
